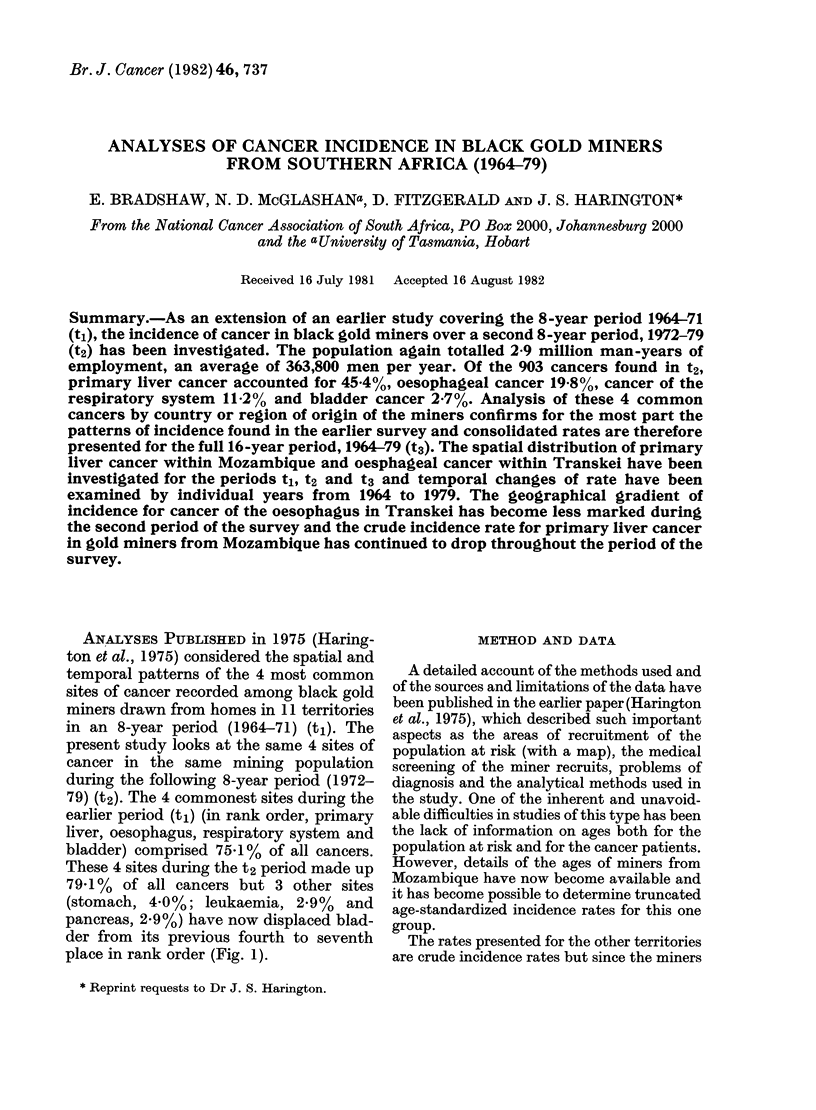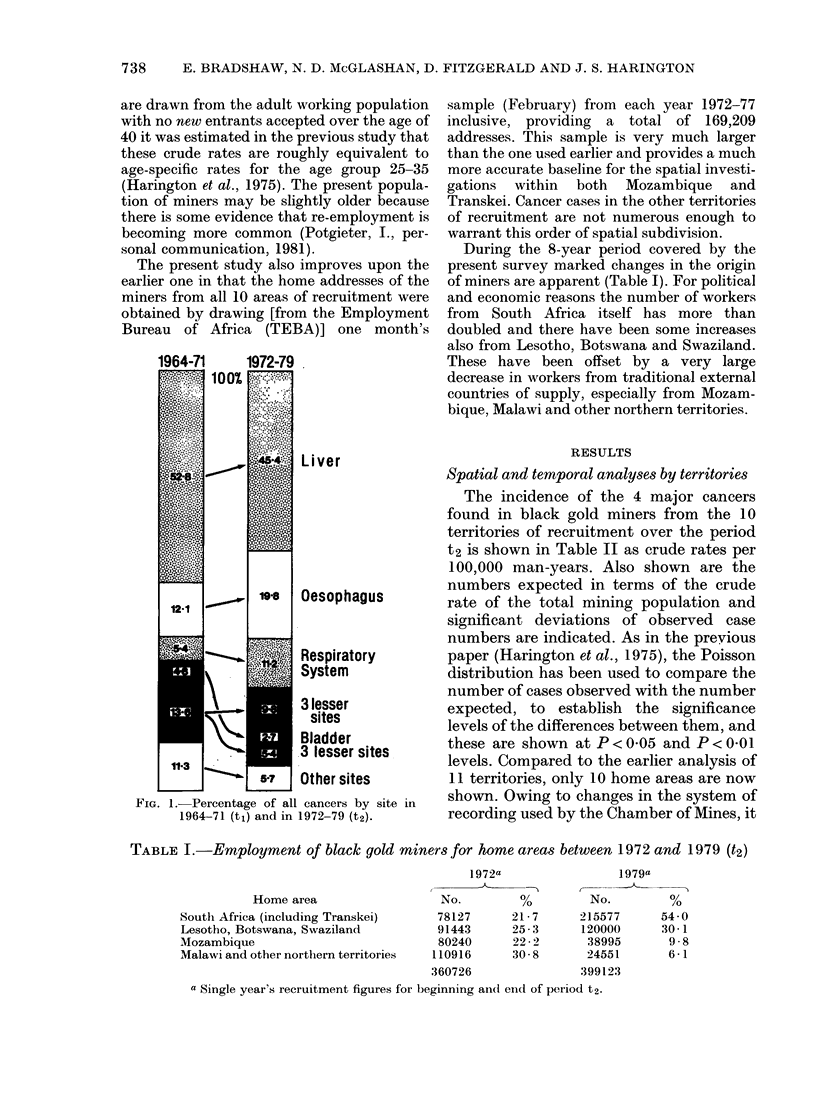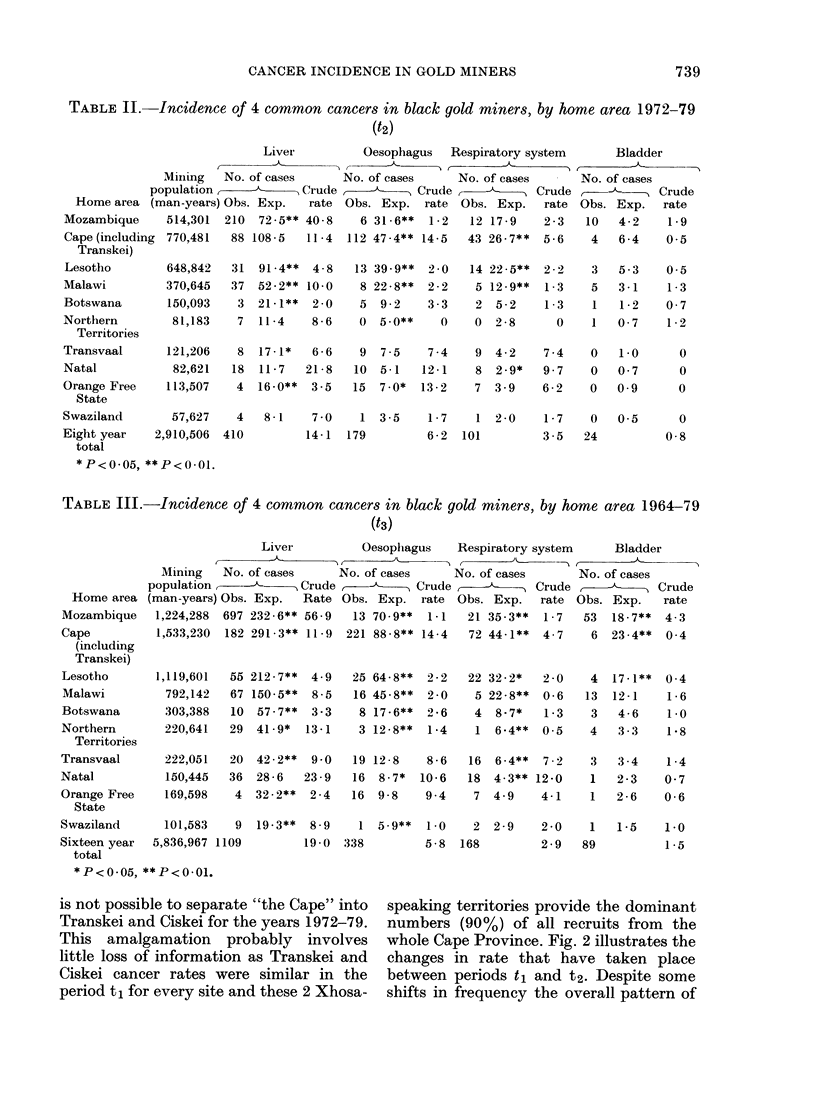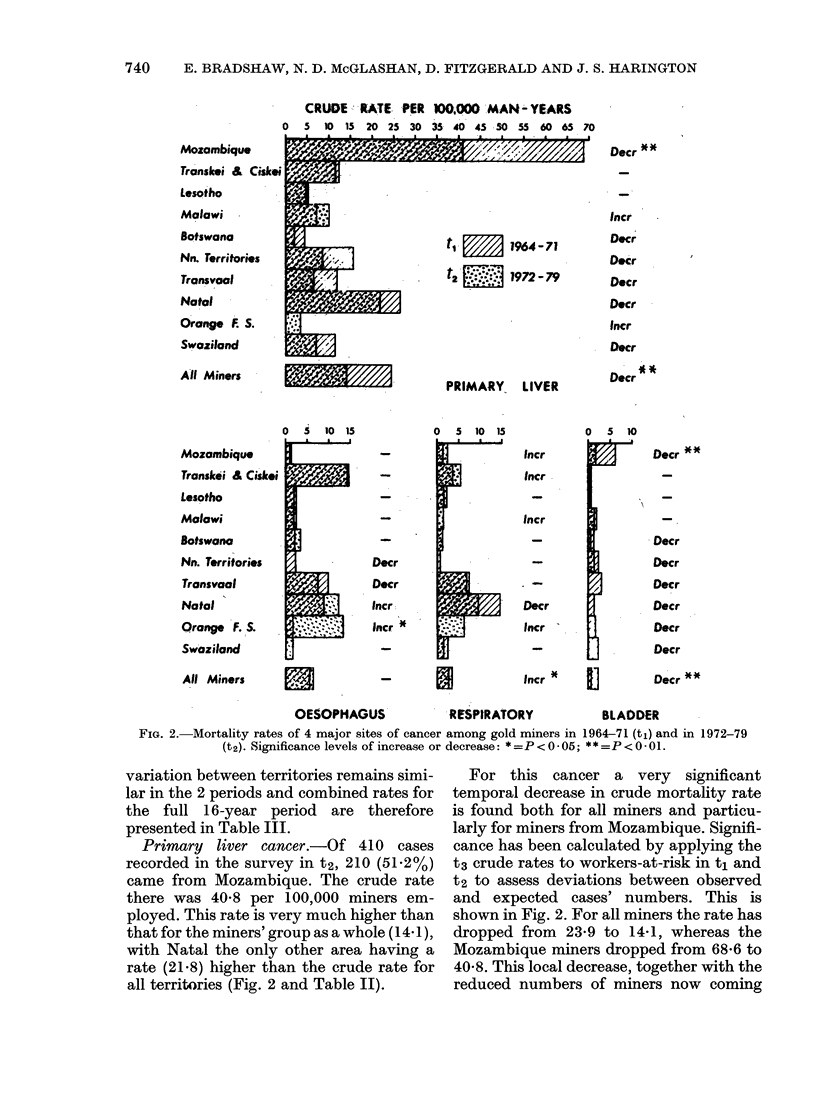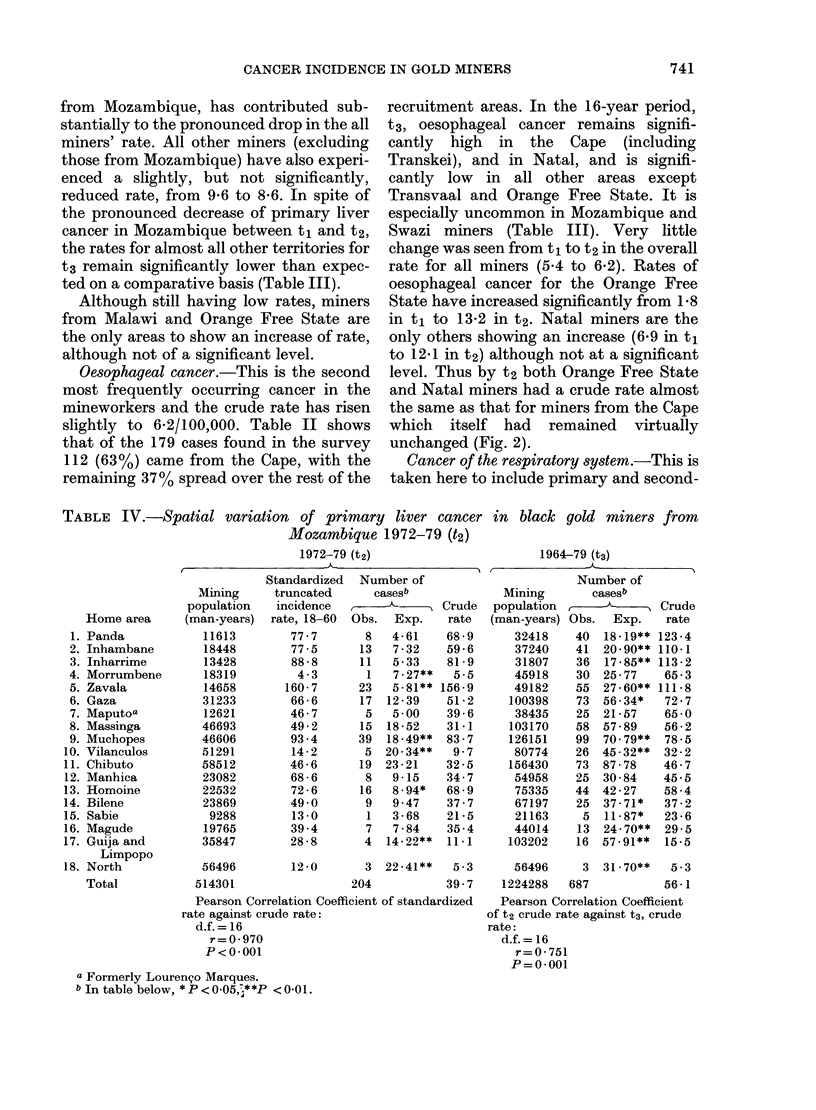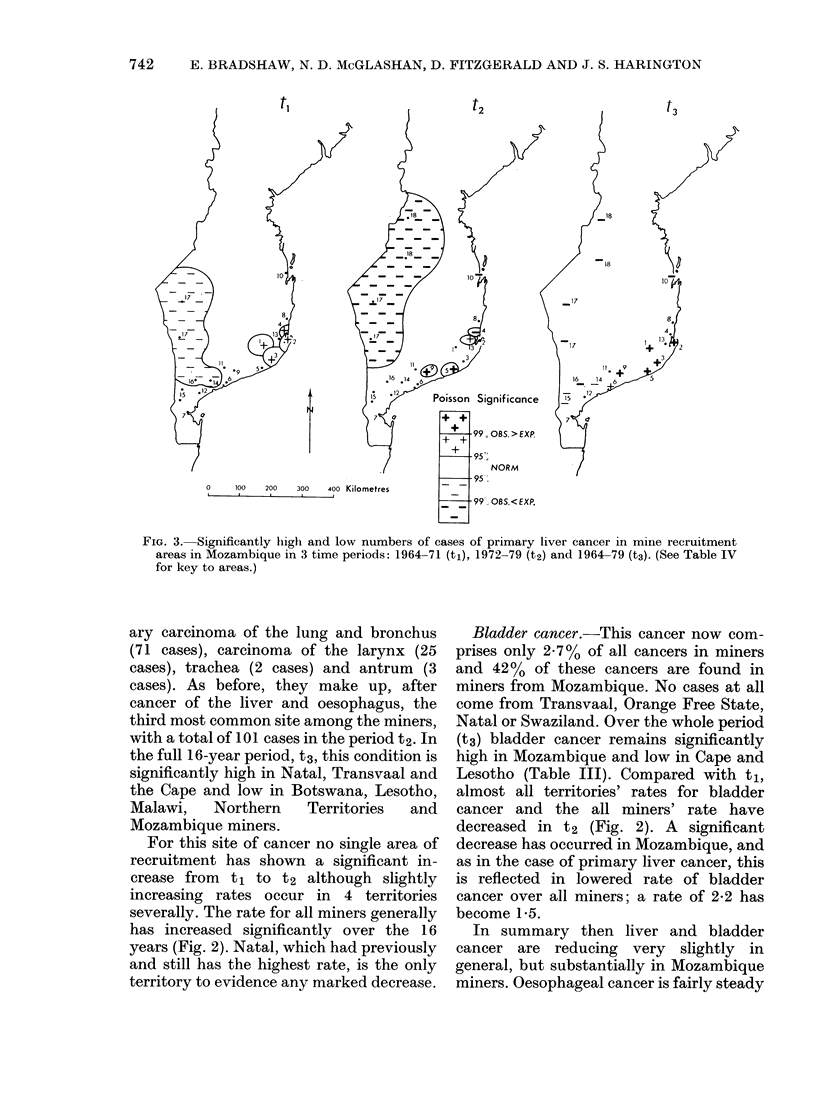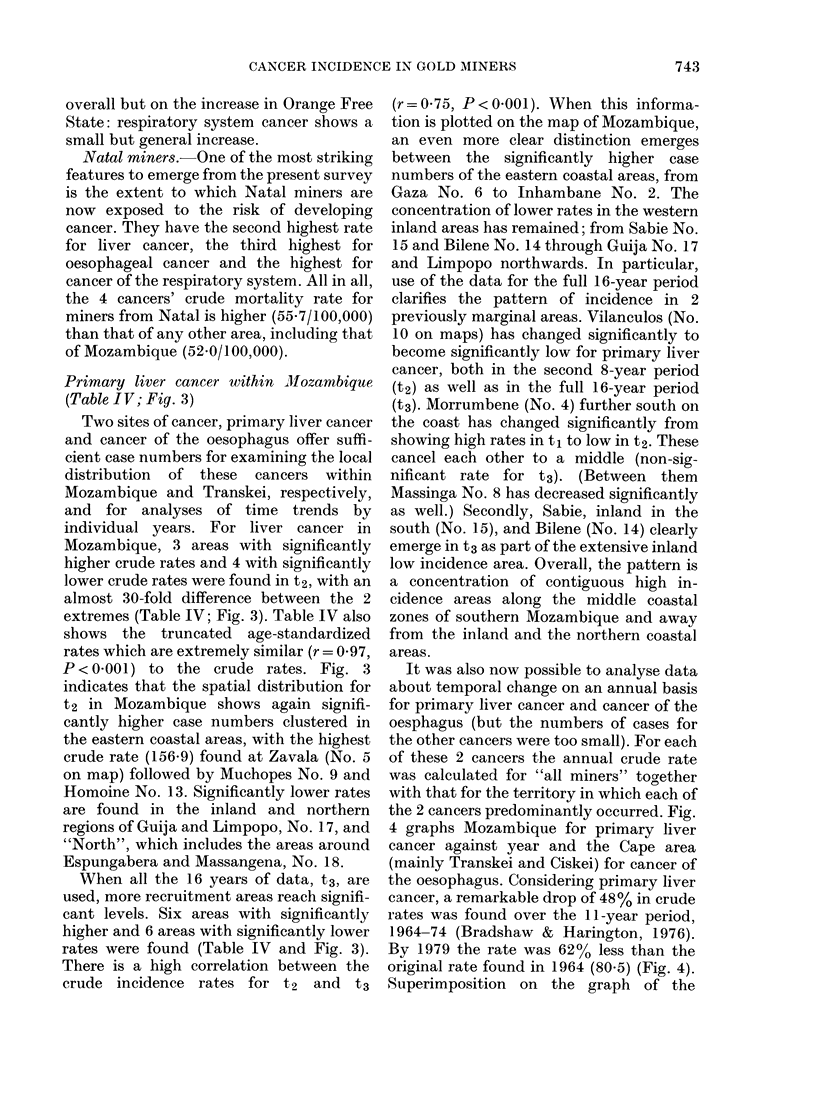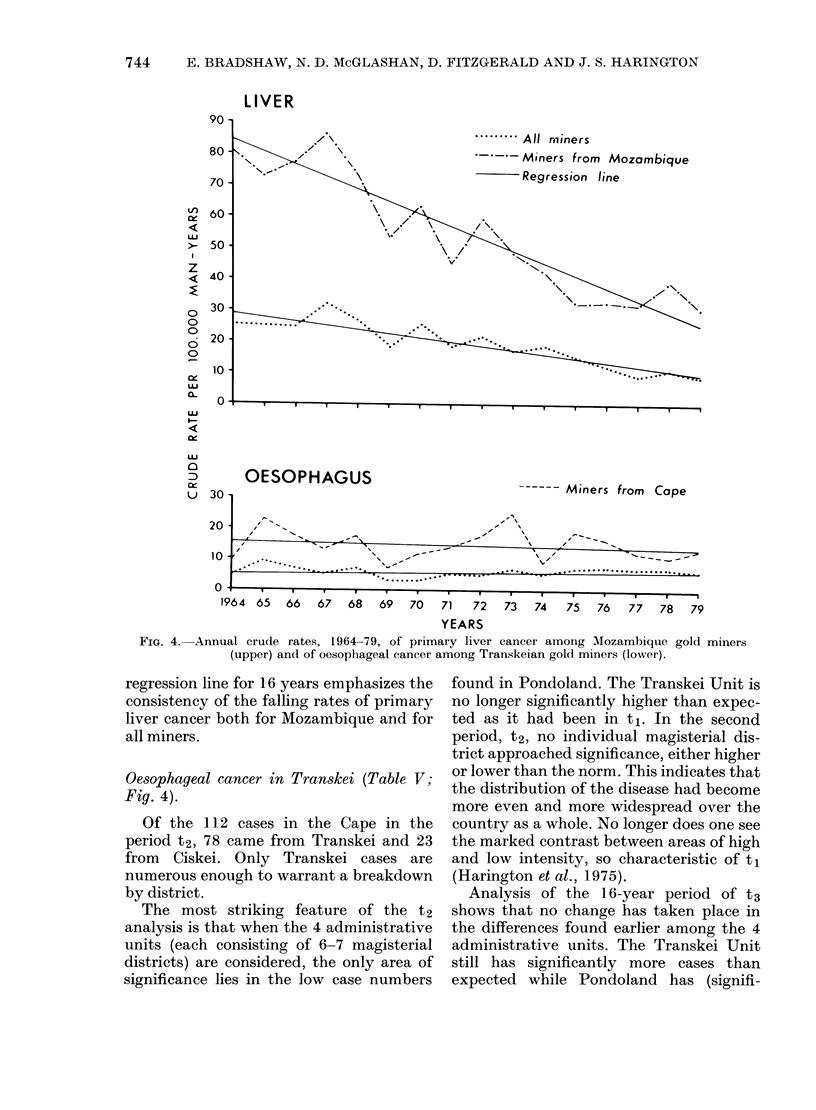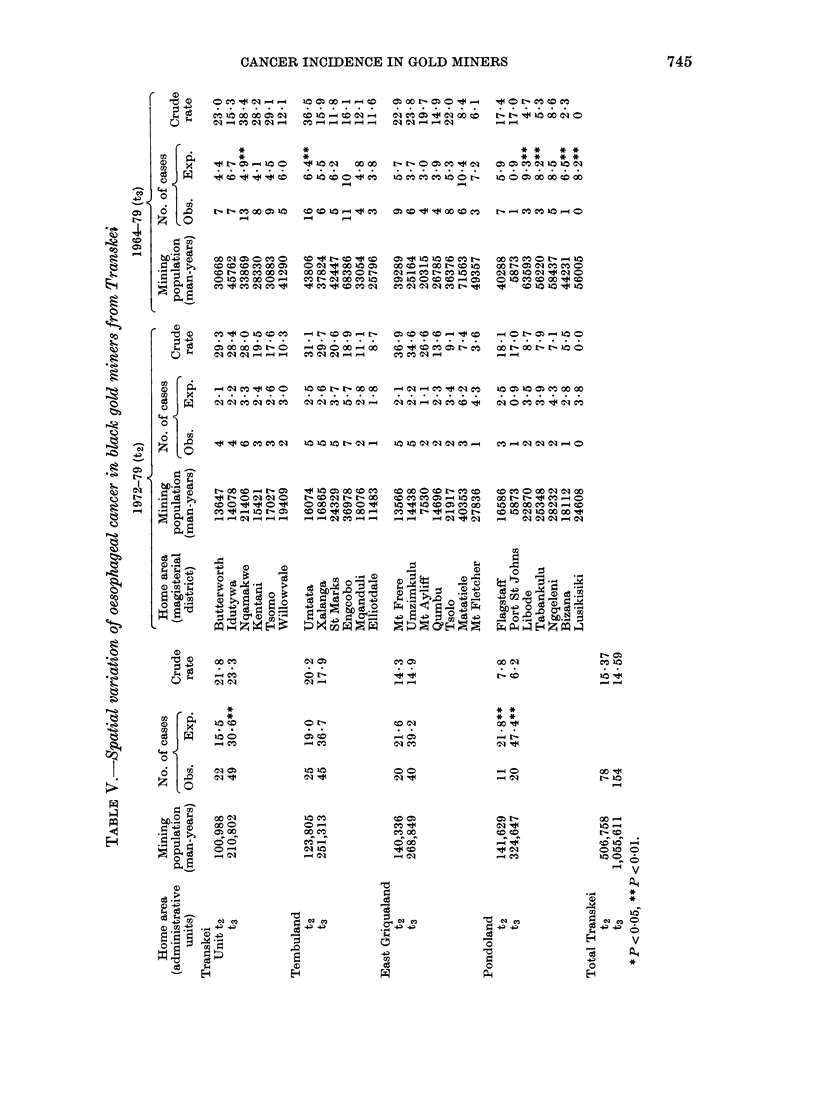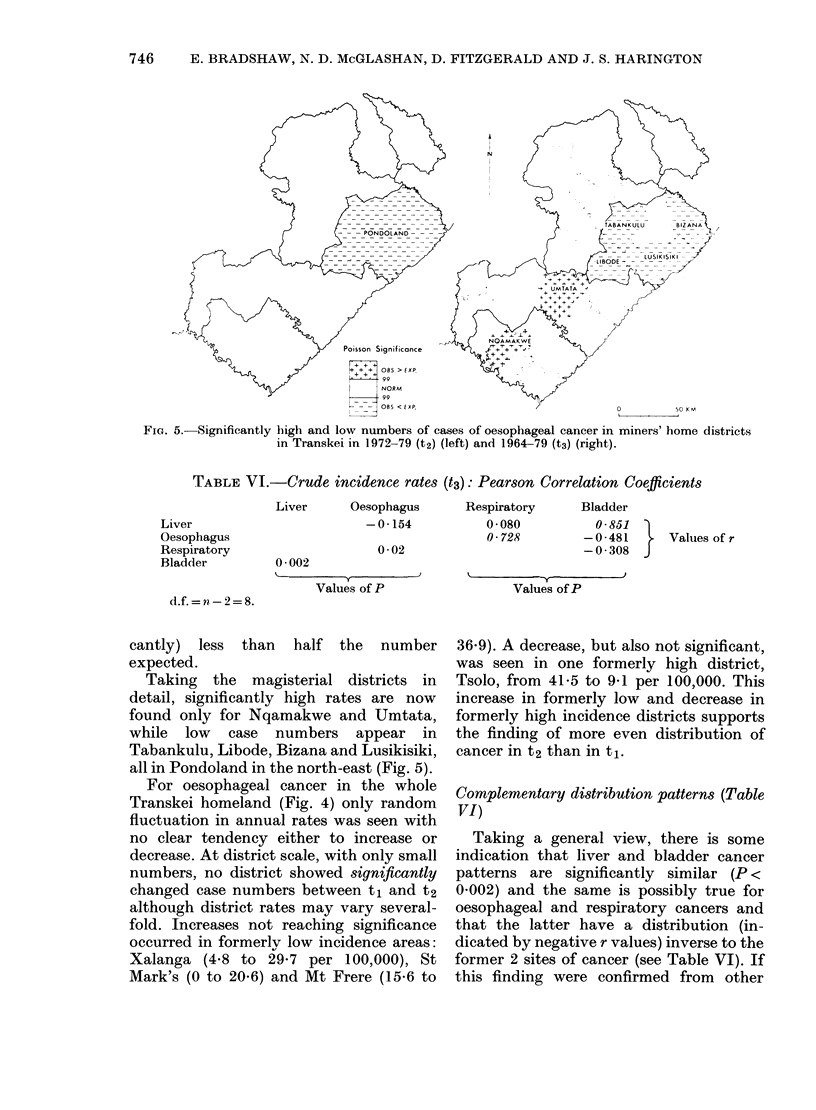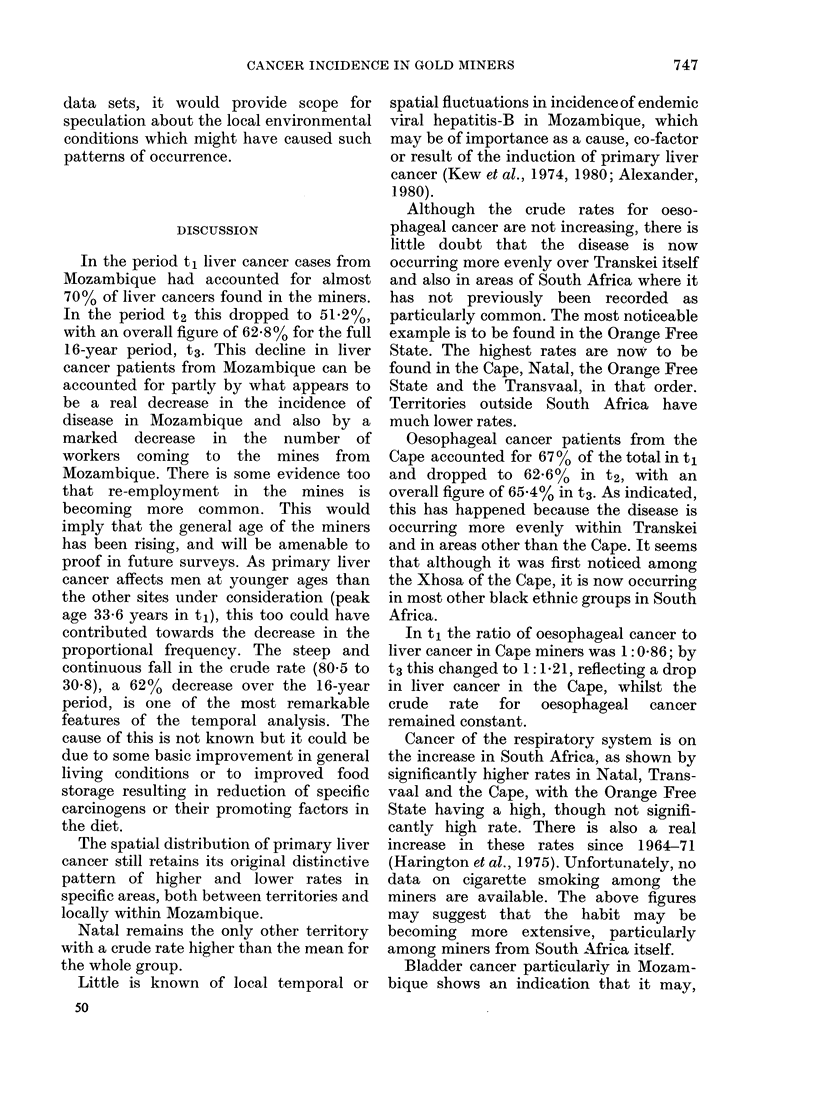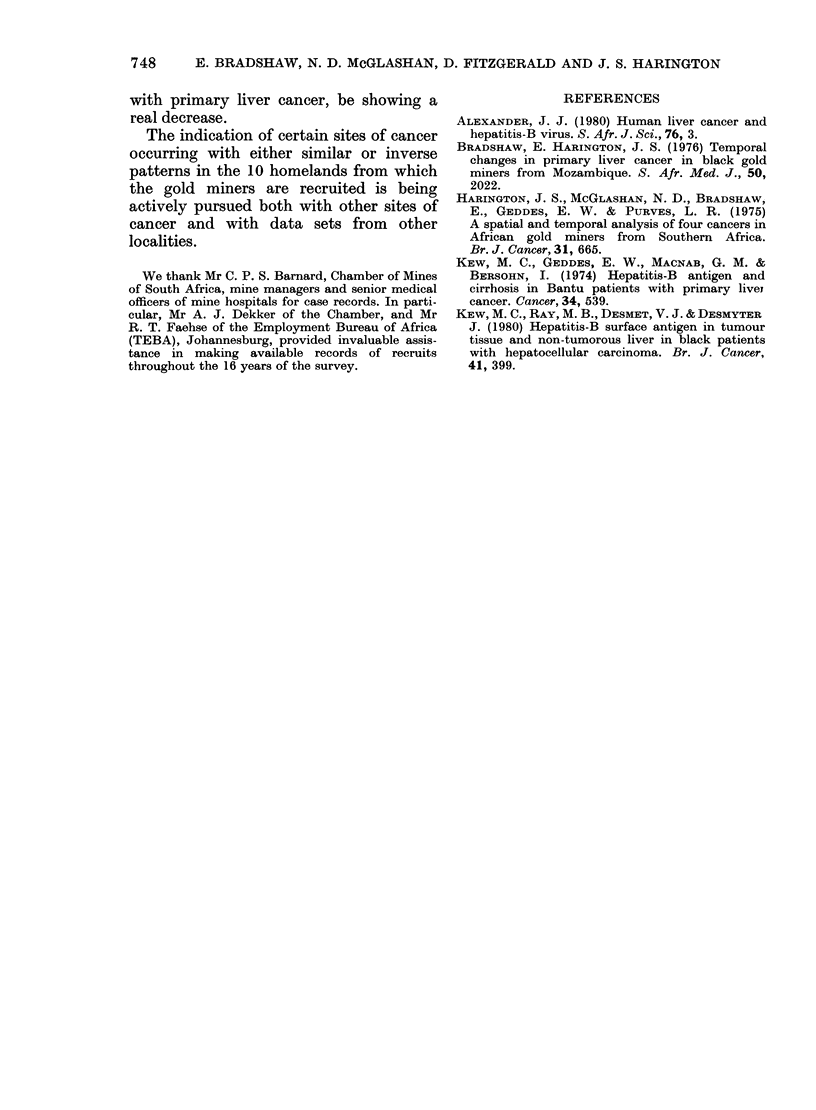# Analyses of cancer incidence in black gold miners from Southern Africa (1964-79).

**DOI:** 10.1038/bjc.1982.266

**Published:** 1982-11

**Authors:** E. Bradshaw, N. D. McGlashan, D. Fitzgerald, J. S. Harington

## Abstract

As an extension of an earlier study covering the 8-year period 1965-71 (t1), the incidence of cancer in black gold miners over a second 8-year period, 1972-79 (t2) has been investigated. The population again totalled 2.9 million man-years of employment, an average of 363,800 men per year. Of the 903 cancers found in t2, primary liver cancer accounted for 45.4%, oesophageal cancer 19.8%, cancer of the respiratory system 11.2% and bladder cancer 2.7%. Analysis of these 4 common cancers by country or region of origin of the miners confirms for the most part the patterns of incidence found in the earlier survey and consolidated rates are therefore presented for the full 16-year period, 1964-79 (t3). The spatial distribution of primary liver cancer within Mozambique and oesphageal cancer within Transkei have been investigated for the periods t1, t2 and t3 and temporal changes of rate have been examined by individual years from 1964 to 1979. The geographical gradient of incidence for cancer of the oesophagus in Transkei has become less marked during the second period of the survey and the crude incidence rate for primary liver cancer in gold miners from Mozambique has continued to drop throughout the period of the survey.


*Br. J. Cancer* (1982) **46**, 737

### ANALYSES OF CANCER INCIDENCE IN BLACK GOLD MINERS FROM SOUTHERN AFRICA (1964–79)

E. BRADSHAW, N. D. McGLASHAN[a], D. FITZGERALD AND J. S. HARINGTON*

*From the National Cancer Association of South Africa, PO Box 2000, Johannesburg 2000 and the [a]University of Tasmania, Hobart*

Received 16 July 1981 Accepted 16 August 1982

**Summary.—As an extension of an earlier study covering the 8-year period 1964–71 ($t_1$), the incidence of cancer in black gold miners over a second 8-year period, 1972–79 ($t_2$) has been investigated. The population again totalled 2·9 million man-years of employment, an average of 363,800 men per year. Of the 903 cancers found in $t_2$, primary liver cancer accounted for 45·4%, oesophageal cancer 19·8%, cancer of the respiratory system 11·2% and bladder cancer 2·7%. Analysis of these 4 common cancers by country or region of origin of the miners confirms for the most part the patterns of incidence found in the earlier survey and consolidated rates are therefore presented for the full 16-year period, 1964–79 ($t_3$). The spatial distribution of primary liver cancer within Mozambique and oesphageal cancer within Transkei have been investigated for the periods $t_1$, $t_2$ and $t_3$ and temporal changes of rate have been examined by individual years from 1964 to 1979. The geographical gradient of incidence for cancer of the oesophagus in Transkei has become less marked during the second period of the survey and the crude incidence rate for primary liver cancer in gold miners from Mozambique has continued to drop throughout the period of the survey.**

ANALYSES PUBLISHED in 1975 (Harington *et al.*, 1975) considered the spatial and temporal patterns of the 4 most common sites of cancer recorded among black gold miners drawn from homes in 11 territories in an 8-year period (1964–71) ($t_1$). The present study looks at the same 4 sites of cancer in the same mining population during the following 8-year period (1972–79) ($t_2$). The 4 commonest sites during the earlier period ($t_1$) (in rank order, primary liver, oesophagus, respiratory system and bladder) comprised 75·1% of all cancers. These 4 sites during the $t_2$ period made up 79·1% of all cancers but 3 other sites (stomach, 4·0%; leukaemia, 2·9% and pancreas, 2·9%) have now displaced bladder from its previous fourth to seventh place in rank order (Fig. 1).

### METHOD AND DATA

A detailed account of the methods used and of the sources and limitations of the data have been published in the earlier paper (Harington *et al.*, 1975), which described such important aspects as the areas of recruitment of the population at risk (with a map), the medical screening of the miner recruits, problems of diagnosis and the analytical methods used in the study. One of the inherent and unavoidable difficulties in studies of this type has been the lack of information on ages both for the population at risk and for the cancer patients. However, details of the ages of miners from Mozambique have now become available and it has become possible to determine truncated age-standardized incidence rates for this one group.

The rates presented for the other territories are crude incidence rates but since the miners

* Reprint requests to Dr J. S. Harington.

are drawn from the adult working population with no *new* entrants accepted over the age of 40 it was estimated in the previous study that these crude rates are roughly equivalent to age-specific rates for the age group 25–35 (Harington *et al.*, 1975). The present population of miners may be slightly older because there is some evidence that re-employment is becoming more common (Potgieter, I., personal communication, 1981).

The present study also improves upon the earlier one in that the home addresses of the miners from all 10 areas of recruitment were obtained by drawing [from the Employment Bureau of Africa (TEBA)] one month's sample (February) from each year 1972–77 inclusive, providing a total of 169,209 addresses. This sample is very much larger than the one used earlier and provides a much more accurate baseline for the spatial investigations within both Mozambique and Transkei. Cancer cases in the other territories of recruitment are not numerous enough to warrant this order of spatial subdivision.

FIG. 1.—Percentage of all cancers by site in 1964–71 ($t_1$) and in 1972–79 ($t_2$).

During the 8-year period covered by the present survey marked changes in the origin of miners are apparent (Table I). For political and economic reasons the number of workers from South Africa itself has more than doubled and there have been some increases also from Lesotho, Botswana and Swaziland. These have been offset by a very large decrease in workers from traditional external countries of supply, especially from Mozambique, Malawi and other northern territories.

### RESULTS

### *Spatial and temporal analyses by territories*

The incidence of the 4 major cancers found in black gold miners from the 10 territories of recruitment over the period $t_2$ is shown in Table II as crude rates per 100,000 man-years. Also shown are the numbers expected in terms of the crude rate of the total mining population and significant deviations of observed case numbers are indicated. As in the previous paper (Harington *et al.*, 1975), the Poisson distribution has been used to compare the number of cases observed with the number expected, to establish the significance levels of the differences between them, and these are shown at $P < 0{\cdot}05$ and $P < 0{\cdot}01$ levels. Compared to the earlier analysis of 11 territories, only 10 home areas are now shown. Owing to changes in the system of recording used by the Chamber of Mines, it

TABLE I.—*Employment of black gold miners for home areas between* 1972 *and* 1979 ($t_2$)

| | 1972[a] | | 1979[a] | |
|---|---|---|---|---|
| Home area | No. | % | No. | % |
| South Africa (including Transkei) | 78127 | 21·7 | 215577 | 54·0 |
| Lesotho, Botswana, Swaziland | 91443 | 25·3 | 120000 | 30·1 |
| Mozambique | 80240 | 22·2 | 38995 | 9·8 |
| Malawi and other northern territories | 110916 | 30·8 | 24551 | 6·1 |
| | 360726 | | 399123 | |

[a] Single year's recruitment figures for beginning and end of period $t_2$.

TABLE II.—*Incidence of 4 common cancers in black gold miners, by home area 1972–79 ($t_2$)*

| | | Liver | | | Oesophagus | | | Respiratory system | | | Bladder | | |
|---|---|---|---|---|---|---|---|---|---|---|---|---|---|
| | Mining population | No. of cases | | Crude | No. of cases | | Crude | No. of cases | | Crude | No. of cases | | Crude |
| Home area | (man-years) | Obs. | Exp. | rate | Obs. | Exp. | rate | Obs. | Exp. | rate | Obs. | Exp. | rate |
| Mozambique | 514,301 | 210 | 72·5** | 40·8 | 6 | 31·6** | 1·2 | 12 | 17·9 | 2·3 | 10 | 4·2 | 1·9 |
| Cape (including Transkei) | 770,481 | 88 | 108·5 | 11·4 | 112 | 47·4** | 14·5 | 43 | 26·7** | 5·6 | 4 | 6·4 | 0·5 |
| Lesotho | 648,842 | 31 | 91·4** | 4·8 | 13 | 39·9** | 2·0 | 14 | 22·5** | 2·2 | 3 | 5·3 | 0·5 |
| Malawi | 370,645 | 37 | 52·2** | 10·0 | 8 | 22·8** | 2·2 | 5 | 12·9** | 1·3 | 5 | 3·1 | 1·3 |
| Botswana | 150,093 | 3 | 21·1** | 2·0 | 5 | 9·2 | 3·3 | 2 | 5·2 | 1·3 | 1 | 1·2 | 0·7 |
| Northern Territories | 81,183 | 7 | 11·4 | 8·6 | 0 | 5·0** | 0 | 0 | 2·8 | 0 | 1 | 0·7 | 1·2 |
| Transvaal | 121,206 | 8 | 17·1* | 6·6 | 9 | 7·5 | 7·4 | 9 | 4·2 | 7·4 | 0 | 1·0 | 0 |
| Natal | 82,621 | 18 | 11·7 | 21·8 | 10 | 5·1 | 12·1 | 8 | 2·9* | 9·7 | 0 | 0·7 | 0 |
| Orange Free State | 113,507 | 4 | 16·0** | 3·5 | 15 | 7·0* | 13·2 | 7 | 3·9 | 6·2 | 0 | 0·9 | 0 |
| Swaziland | 57,627 | 4 | 8·1 | 7·0 | 1 | 3·5 | 1·7 | 1 | 2·0 | 1·7 | 0 | 0·5 | 0 |
| Eight year total | 2,910,506 | 410 | | 14·1 | 179 | | 6·2 | 101 | | 3·5 | 24 | | 0·8 |

* $P<0·05$, ** $P<0·01$.

TABLE III.—*Incidence of 4 common cancers in black gold miners, by home area 1964–79 ($t_3$)*

| | | Liver | | | Oesophagus | | | Respiratory system | | | Bladder | | |
|---|---|---|---|---|---|---|---|---|---|---|---|---|---|
| | Mining population | No. of cases | | Crude | No. of cases | | Crude | No. of cases | | Crude | No. of cases | | Crude |
| Home area | (man-years) | Obs. | Exp. | Rate | Obs. | Exp. | rate | Obs. | Exp. | rate | Obs. | Exp. | rate |
| Mozambique | 1,224,288 | 697 | 232·6** | 56·9 | 13 | 70·9** | 1·1 | 21 | 35·3** | 1·7 | 53 | 18·7** | 4·3 |
| Cape (including Transkei) | 1,533,230 | 182 | 291·3** | 11·9 | 221 | 88·8** | 14·4 | 72 | 44·1** | 4·7 | 6 | 23·4** | 0·4 |
| Lesotho | 1,119,601 | 55 | 212·7** | 4·9 | 25 | 64·8** | 2·2 | 22 | 32·2* | 2·0 | 4 | 17·1** | 0·4 |
| Malawi | 792,142 | 67 | 150·5** | 8·5 | 16 | 45·8** | 2·0 | 5 | 22·8** | 0·6 | 13 | 12·1 | 1·6 |
| Botswana | 303,388 | 10 | 57·7** | 3·3 | 8 | 17·6** | 2·6 | 4 | 8·7* | 1·3 | 3 | 4·6 | 1·0 |
| Northern Territories | 220,641 | 29 | 41·9* | 13·1 | 3 | 12·8** | 1·4 | 1 | 6·4** | 0·5 | 4 | 3·3 | 1·8 |
| Transvaal | 222,051 | 20 | 42·2** | 9·0 | 19 | 12·8 | 8·6 | 16 | 6·4** | 7·2 | 3 | 3·4 | 1·4 |
| Natal | 150,445 | 36 | 28·6 | 23·9 | 16 | 8·7* | 10·6 | 18 | 4·3** | 12·0 | 1 | 2·3 | 0·7 |
| Orange Free State | 169,598 | 4 | 32·2** | 2·4 | 16 | 9·8 | 9·4 | 7 | 4·9 | 4·1 | 1 | 2·6 | 0·6 |
| Swaziland | 101,583 | 9 | 19·3** | 8·9 | 1 | 5·9** | 1·0 | 2 | 2·9 | 2·0 | 1 | 1·5 | 1·0 |
| Sixteen year total | 5,836,967 | 1109 | | 19·0 | 338 | | 5·8 | 168 | | 2·9 | 89 | | 1·5 |

* $P<0·05$, ** $P<0·01$.

is not possible to separate "the Cape" into Transkei and Ciskei for the years 1972–79. This amalgamation probably involves little loss of information as Transkei and Ciskei cancer rates were similar in the period $t_1$ for every site and these 2 Xhosa-speaking territories provide the dominant numbers (90%) of all recruits from the whole Cape Province. Fig. 2 illustrates the changes in rate that have taken place between periods $t_1$ and $t_2$. Despite some shifts in frequency the overall pattern of

FIG. 2.—Mortality rates of 4 major sites of cancer among gold miners in 1964–71 ($t_1$) and in 1972–79 ($t_2$). Significance levels of increase or decrease: $* = P < 0 \cdot 05$; $** = P < 0 \cdot 01$.

variation between territories remains similar in the 2 periods and combined rates for the full 16-year period are therefore presented in Table III.

*Primary liver cancer.*—Of 410 cases recorded in the survey in $t_2$, 210 (51·2%) came from Mozambique. The crude rate there was 40·8 per 100,000 miners employed. This rate is very much higher than that for the miners' group as a whole (14·1), with Natal the only other area having a rate (21·8) higher than the crude rate for all territories (Fig. 2 and Table II).

For this cancer a very significant temporal decrease in crude mortality rate is found both for all miners and particularly for miners from Mozambique. Significance has been calculated by applying the $t_3$ crude rates to workers-at-risk in $t_1$ and $t_2$ to assess deviations between observed and expected cases' numbers. This is shown in Fig. 2. For all miners the rate has dropped from 23·9 to 14·1, whereas the Mozambique miners dropped from 68·6 to 40·8. This local decrease, together with the reduced numbers of miners now coming from Mozambique, has contributed substantially to the pronounced drop in the all miners' rate. All other miners (excluding those from Mozambique) have also experienced a slightly, but not significantly, reduced rate, from 9·6 to 8·6. In spite of the pronounced decrease of primary liver cancer in Mozambique between $t_1$ and $t_2$, the rates for almost all other territories for $t_3$ remain significantly lower than expected on a comparative basis (Table III).

Although still having low rates, miners from Malawi and Orange Free State are the only areas to show an increase of rate, although not of a significant level.

*Oesophageal cancer.*—This is the second most frequently occurring cancer in the mineworkers and the crude rate has risen slightly to 6·2/100,000. Table II shows that of the 179 cases found in the survey 112 (63%) came from the Cape, with the remaining 37% spread over the rest of the recruitment areas. In the 16-year period, $t_3$, oesophageal cancer remains significantly high in the Cape (including Transkei), and in Natal, and is significantly low in all other areas except Transvaal and Orange Free State. It is especially uncommon in Mozambique and Swazi miners (Table III). Very little change was seen from $t_1$ to $t_2$ in the overall rate for all miners (5·4 to 6·2). Rates of oesophageal cancer for the Orange Free State have increased significantly from 1·8 in $t_1$ to 13·2 in $t_2$. Natal miners are the only others showing an increase (6·9 in $t_1$ to 12·1 in $t_2$) although not at a significant level. Thus by $t_2$ both Orange Free State and Natal miners had a crude rate almost the same as that for miners from the Cape which itself had remained virtually unchanged (Fig. 2).

*Cancer of the respiratory system.*—This is taken here to include primary and second-

TABLE IV.—*Spatial variation of primary liver cancer in black gold miners from Mozambique* 1972–79 ($t_2$)

| | 1972–79 ($t_2$) | | | | | 1964–79 ($t_3$) | | | |
|---|---|---|---|---|---|---|---|---|---|
| | Mining population (man-years) | Standardized truncated incidence rate, 18–60 | Number of cases[b] | | Crude rate | Mining population (man-years) | Number of cases[b] | | Crude rate |
| Home area | | | Obs. | Exp. | | | Obs. | Exp. | |
| 1. Panda | 11613 | 77·7 | 8 | 4·61 | 68·9 | 32418 | 40 | 18·19** | 123·4 |
| 2. Inhambane | 18448 | 77·5 | 13 | 7·32 | 59·6 | 37240 | 41 | 20·90** | 110·1 |
| 3. Inharrime | 13428 | 88·8 | 11 | 5·33 | 81·9 | 31807 | 36 | 17·85** | 113·2 |
| 4. Morrumbene | 18319 | 4·3 | 1 | 7·27** | 5·5 | 45918 | 30 | 25·77 | 65·3 |
| 5. Zavala | 14658 | 160·7 | 23 | 5·81** | 156·9 | 49182 | 55 | 27·60** | 111·8 |
| 6. Gaza | 31233 | 66·6 | 17 | 12·39 | 51·2 | 100398 | 73 | 56·34* | 72·7 |
| 7. Maputo[a] | 12621 | 46·7 | 5 | 5·00 | 39·6 | 38435 | 25 | 21·57 | 65·0 |
| 8. Massinga | 46693 | 49·2 | 15 | 18·52 | 31·1 | 103170 | 58 | 57·89 | 56·2 |
| 9. Muchopes | 46606 | 93·4 | 39 | 18·49** | 83·7 | 126151 | 99 | 70·79** | 78·5 |
| 10. Vilanculos | 51291 | 14·2 | 5 | 20·34** | 9·7 | 80774 | 26 | 45·32** | 32·2 |
| 11. Chibuto | 58512 | 46·6 | 19 | 23·21 | 32·5 | 156430 | 73 | 87·78 | 46·7 |
| 12. Manhica | 23082 | 68·6 | 8 | 9·15 | 34·7 | 54958 | 25 | 30·84 | 45·5 |
| 13. Homoine | 22532 | 72·6 | 16 | 8·94* | 68·9 | 75335 | 44 | 42·27 | 58·4 |
| 14. Bilene | 23869 | 49·0 | 9 | 9·47 | 37·7 | 67197 | 25 | 37·71* | 37·2 |
| 15. Sabie | 9288 | 13·0 | 1 | 3·68 | 21·5 | 21163 | 5 | 11·87* | 23·6 |
| 16. Magude | 19765 | 39·4 | 7 | 7·84 | 35·4 | 44014 | 13 | 24·70** | 29·5 |
| 17. Guija and Limpopo | 35847 | 28·8 | 4 | 14·22** | 11·1 | 103202 | 16 | 57·91** | 15·5 |
| 18. North | 56496 | 12·0 | 3 | 22·41** | 5·3 | 56496 | 3 | 31·70** | 5·3 |
| Total | 514301 | | 204 | | 39·7 | 1224288 | 687 | | 56·1 |

Pearson Correlation Coefficient of standardized rate against crude rate:
d.f. = 16
$r = 0{\cdot}970$
$P < 0{\cdot}001$

Pearson Correlation Coefficient of $t_2$ crude rate against $t_3$, crude rate:
d.f. = 16
$r = 0{\cdot}751$
$P = 0{\cdot}001$

[a] Formerly Lourenço Marques.
[b] In table below, $*P < 0{\cdot}05$, $**P < 0{\cdot}01$.

FIG. 3.—Significantly high and low numbers of cases of primary liver cancer in mine recruitment areas in Mozambique in 3 time periods: 1964–71 ($t_1$), 1972–79 ($t_2$) and 1964–79 ($t_3$). (See Table IV for key to areas.)

ary carcinoma of the lung and bronchus (71 cases), carcinoma of the larynx (25 cases), trachea (2 cases) and antrum (3 cases). As before, they make up, after cancer of the liver and oesophagus, the third most common site among the miners, with a total of 101 cases in the period $t_2$. In the full 16-year period, $t_3$, this condition is significantly high in Natal, Transvaal and the Cape and low in Botswana, Lesotho, Malawi, Northern Territories and Mozambique miners.

For this site of cancer no single area of recruitment has shown a significant increase from $t_1$ to $t_2$ although slightly increasing rates occur in 4 territories severally. The rate for all miners generally has increased significantly over the 16 years (Fig. 2). Natal, which had previously and still has the highest rate, is the only territory to evidence any marked decrease.

*Bladder cancer.*—This cancer now comprises only 2·7% of all cancers in miners and 42% of these cancers are found in miners from Mozambique. No cases at all come from Transvaal, Orange Free State, Natal or Swaziland. Over the whole period ($t_3$) bladder cancer remains significantly high in Mozambique and low in Cape and Lesotho (Table III). Compared with $t_1$, almost all territories' rates for bladder cancer and the all miners' rate have decreased in $t_2$ (Fig. 2). A significant decrease has occurred in Mozambique, and as in the case of primary liver cancer, this is reflected in lowered rate of bladder cancer over all miners; a rate of 2·2 has become 1·5.

In summary then liver and bladder cancer are reducing very slightly in general, but substantially in Mozambique miners. Oesophageal cancer is fairly steady overall but on the increase in Orange Free State: respiratory system cancer shows a small but general increase.

*Natal miners*.—One of the most striking features to emerge from the present survey is the extent to which Natal miners are now exposed to the risk of developing cancer. They have the second highest rate for liver cancer, the third highest for oesophageal cancer and the highest for cancer of the respiratory system. All in all, the 4 cancers' crude mortality rate for miners from Natal is higher (55·7/100,000) than that of any other area, including that of Mozambique (52·0/100,000).

*Primary liver cancer within Mozambique (Table IV; Fig.* 3)

Two sites of cancer, primary liver cancer and cancer of the oesophagus offer sufficient case numbers for examining the local distribution of these cancers within Mozambique and Transkei, respectively, and for analyses of time trends by individual years. For liver cancer in Mozambique, 3 areas with significantly higher crude rates and 4 with significantly lower crude rates were found in $t_2$, with an almost 30-fold difference between the 2 extremes (Table IV; Fig. 3). Table IV also shows the truncated age-standardized rates which are extremely similar ($r = 0{\cdot}97$, $P < 0{\cdot}001$) to the crude rates. Fig. 3 indicates that the spatial distribution for $t_2$ in Mozambique shows again significantly higher case numbers clustered in the eastern coastal areas, with the highest crude rate (156·9) found at Zavala (No. 5 on map) followed by Muchopes No. 9 and Homoine No. 13. Significantly lower rates are found in the inland and northern regions of Guija and Limpopo, No. 17, and "North", which includes the areas around Espungabera and Massangena, No. 18.

When all the 16 years of data, $t_3$, are used, more recruitment areas reach significant levels. Six areas with significantly higher and 6 areas with significantly lower rates were found (Table IV and Fig. 3). There is a high correlation between the crude incidence rates for $t_2$ and $t_3$ ($r = 0{\cdot}75$, $P < 0{\cdot}001$). When this information is plotted on the map of Mozambique, an even more clear distinction emerges between the significantly higher case numbers of the eastern coastal areas, from Gaza No. 6 to Inhambane No. 2. The concentration of lower rates in the western inland areas has remained; from Sabie No. 15 and Bilene No. 14 through Guija No. 17 and Limpopo northwards. In particular, use of the data for the full 16-year period clarifies the pattern of incidence in 2 previously marginal areas. Vilanculos (No. 10 on maps) has changed significantly to become significantly low for primary liver cancer, both in the second 8-year period ($t_2$) as well as in the full 16-year period ($t_3$). Morrumbene (No. 4) further south on the coast has changed significantly from showing high rates in $t_1$ to low in $t_2$. These cancel each other to a middle (non-significant rate for $t_3$). (Between them Massinga No. 8 has decreased significantly as well.) Secondly, Sabie, inland in the south (No. 15), and Bilene (No. 14) clearly emerge in $t_3$ as part of the extensive inland low incidence area. Overall, the pattern is a concentration of contiguous high incidence areas along the middle coastal zones of southern Mozambique and away from the inland and the northern coastal areas.

It was also now possible to analyse data about temporal change on an annual basis for primary liver cancer and cancer of the oesphagus (but the numbers of cases for the other cancers were too small). For each of these 2 cancers the annual crude rate was calculated for "all miners" together with that for the territory in which each of the 2 cancers predominantly occurred. Fig. 4 graphs Mozambique for primary liver cancer against year and the Cape area (mainly Transkei and Ciskei) for cancer of the oesophagus. Considering primary liver cancer, a remarkable drop of 48% in crude rates was found over the 11-year period, 1964–74 (Bradshaw & Harington, 1976). By 1979 the rate was 62% less than the original rate found in 1964 (80·5) (Fig. 4). Superimposition on the graph of the

FIG. 4.—Annual crude rates, 1964–79, of primary liver cancer among Mozambique gold miners (upper) and of oesophageal cancer among Transkeian gold miners (lower).

regression line for 16 years emphasizes the consistency of the falling rates of primary liver cancer both for Mozambique and for all miners.

*Oesophageal cancer in Transkei* (*Table V; Fig.* 4).

Of the 112 cases in the Cape in the period $t_2$, 78 came from Transkei and 23 from Ciskei. Only Transkei cases are numerous enough to warrant a breakdown by district.

The most striking feature of the $t_2$ analysis is that when the 4 administrative units (each consisting of 6–7 magisterial districts) are considered, the only area of significance lies in the low case numbers found in Pondoland. The Transkei Unit is no longer significantly higher than expected as it had been in $t_1$. In the second period, $t_2$, no individual magisterial district approached significance, either higher or lower than the norm. This indicates that the distribution of the disease had become more even and more widespread over the country as a whole. No longer does one see the marked contrast between areas of high and low intensity, so characteristic of $t_1$ (Harington *et al.*, 1975).

Analysis of the 16-year period of $t_3$ shows that no change has taken place in the differences found earlier among the 4 administrative units. The Transkei Unit still has significantly more cases than expected while Pondoland has (signifi-

TABLE V.—*Spatial variation of oesophageal cancer in black gold miners from Transkei*

| Home area (administrative units) | Mining population (man-years) | No. of cases | | Crude rate | 1972–79 ($t_2$) | | | | | 1964–79 ($t_3$) | | | |
|---|---|---|---|---|---|---|---|---|---|---|---|---|---|
| | | Obs. | Exp. | | Home area (magisterial district) | Mining population (man-years) | No. of cases Obs. | Exp. | Crude rate | Mining population (man-years) | No. of cases Obs. | Exp. | Crude rate |
| Transkei | | | | | | | | | | | | | |
| Unit $t_2$ | 100,988 | 22 | 15·5 | 21·8 | Butterworth | 13647 | 4 | 2·1 | 29·3 | 30668 | 7 | 4·4 | 23·0 |
| $t_3$ | 210,802 | 49 | 30·6** | 23·3 | Idutywa | 14078 | 4 | 2·2 | 28·4 | 45762 | 7 | 6·7 | 15·3 |
| | | | | | Nqamakwe | 21406 | 6 | 3·3 | 28·0 | 33869 | 13 | 4·9** | 38·4 |
| | | | | | Kentani | 15421 | 3 | 2·4 | 19·5 | 28330 | 8 | 4·1 | 28·2 |
| | | | | | Tsomo | 17027 | 3 | 2·6 | 17·6 | 30883 | 9 | 4·5 | 29·1 |
| | | | | | Willowvale | 19409 | 2 | 3·0 | 10·3 | 41290 | 5 | 6·0 | 12·1 |
| Tembuland | | | | | | | | | | | | | |
| $t_2$ | 123,805 | 25 | 19·0 | 20·2 | Umtata | 16074 | 5 | 2·5 | 31·1 | 43806 | 16 | 6·4** | 36·5 |
| $t_3$ | 251,313 | 45 | 36·7 | 17·9 | Xalanga | 16865 | 5 | 2·6 | 29·7 | 37824 | 6 | 5·5 | 15·9 |
| | | | | | St Marks | 24329 | 5 | 3·7 | 20·6 | 42447 | 5 | 6·2 | 11·8 |
| | | | | | Engcobo | 36978 | 7 | 5·7 | 18·9 | 68386 | 11 | 10 | 16·1 |
| | | | | | Mqanduli | 18076 | 2 | 2·8 | 11·1 | 33054 | 4 | 4·8 | 12·1 |
| | | | | | Elliotdale | 11483 | 1 | 1·8 | 8·7 | 25796 | 3 | 3·8 | 11·6 |
| East Griqualand | | | | | | | | | | | | | |
| $t_2$ | 140,336 | 20 | 21·6 | 14·3 | Mt Frere | 13566 | 5 | 2·1 | 36·9 | 39289 | 9 | 5·7 | 22·9 |
| $t_3$ | 268,849 | 40 | 39·2 | 14·9 | Umzimkulu | 14438 | 5 | 2·2 | 34·6 | 25164 | 6 | 3·7 | 23·8 |
| | | | | | Mt Ayliff | 7530 | 2 | 1·1 | 26·6 | 20315 | 4 | 3·0 | 19·7 |
| | | | | | Qumbu | 14696 | 2 | 2·3 | 13·6 | 26785 | 4 | 3·9 | 14·9 |
| | | | | | Tsolo | 21917 | 2 | 3·4 | 9·1 | 36376 | 8 | 5·3 | 22·0 |
| | | | | | Matatiele | 40353 | 3 | 6·2 | 7·4 | 71563 | 6 | 10·4 | 8·4 |
| | | | | | Mt Fletcher | 27836 | 1 | 4·3 | 3·6 | 49357 | 3 | 7·2 | 6·1 |
| Pondoland | | | | | | | | | | | | | |
| $t_2$ | 141,629 | 11 | 21·8** | 7·8 | Flagstaff | 16586 | 3 | 2·5 | 18·1 | 40288 | 7 | 5·9 | 17·4 |
| $t_3$ | 324,647 | 20 | 47·4** | 6·2 | Port St Johns | 5873 | 1 | 0·9 | 17·0 | 5873 | 1 | 0·9 | 17·0 |
| | | | | | Libode | 22870 | 2 | 3·5 | 8·7 | 63593 | 3 | 9·3** | 4·7 |
| | | | | | Tabankulu | 25348 | 2 | 3·9 | 7·9 | 56220 | 3 | 8·2** | 5·3 |
| | | | | | Ngqeleni | 28232 | 2 | 4·3 | 7·1 | 58437 | 5 | 8·5 | 8·6 |
| | | | | | Bizana | 18112 | 1 | 2·8 | 5·5 | 44231 | 1 | 6·5** | 2·3 |
| | | | | | Lusikisiki | 24608 | 0 | 3·8 | 0·0 | 56005 | 0 | 8·2** | 0 |
| Total Transkei | | | | | | | | | | | | | |
| $t_2$ | 506,758 | 78 | | 15·37 | | | | | | | | | |
| $t_3$ | 1,055,611 | 154 | | 14·59 | | | | | | | | | |

$*P < 0{\cdot}05$, $**P < 0{\cdot}01$.

FIG. 5.—Significantly high and low numbers of cases of oesophageal cancer in miners' home districts in Transkei in 1972–79 ($t_2$) (left) and 1964–79 ($t_3$) (right).

TABLE VI.—*Crude incidence rates* ($t_3$)*: Pearson Correlation Coefficients*

| | Liver | Oesophagus | Respiratory | Bladder | |
|---|---|---|---|---|---|
| Liver | | −0·154 | 0·080 | *0·851* | Values of $r$ |
| Oesophagus | | | *0·728* | −0·481 | |
| Respiratory | | 0·02 | | −0·308 | |
| Bladder | 0·002 | | | | |
| | Values of $P$ | | Values of $P$ | | |

d.f. $= n - 2 = 8$.

cantly) less than half the number expected.

Taking the magisterial districts in detail, significantly high rates are now found only for Nqamakwe and Umtata, while low case numbers appear in Tabankulu, Libode, Bizana and Lusikisiki, all in Pondoland in the north-east (Fig. 5).

For oesophageal cancer in the whole Transkei homeland (Fig. 4) only random fluctuation in annual rates was seen with no clear tendency either to increase or decrease. At district scale, with only small numbers, no district showed *significantly* changed case numbers between $t_1$ and $t_2$ although district rates may vary several-fold. Increases not reaching significance occurred in formerly low incidence areas: Xalanga (4·8 to 29·7 per 100,000), St Mark's (0 to 20·6) and Mt Frere (15·6 to 36·9). A decrease, but also not significant, was seen in one formerly high district, Tsolo, from 41·5 to 9·1 per 100,000. This increase in formerly low and decrease in formerly high incidence districts supports the finding of more even distribution of cancer in $t_2$ than in $t_1$.

*Complementary distribution patterns (Table VI)*

Taking a general view, there is some indication that liver and bladder cancer patterns are significantly similar ($P <$ 0·002) and the same is possibly true for oesophageal and respiratory cancers and that the latter have a distribution (indicated by negative $r$ values) inverse to the former 2 sites of cancer (see Table VI). If this finding were confirmed from other data sets, it would provide scope for speculation about the local environmental conditions which might have caused such patterns of occurrence.

### DISCUSSION

In the period $t_1$ liver cancer cases from Mozambique had accounted for almost 70% of liver cancers found in the miners. In the period $t_2$ this dropped to 51·2%, with an overall figure of 62·8% for the full 16-year period, $t_3$. This decline in liver cancer patients from Mozambique can be accounted for partly by what appears to be a real decrease in the incidence of disease in Mozambique and also by a marked decrease in the number of workers coming to the mines from Mozambique. There is some evidence too that re-employment in the mines is becoming more common. This would imply that the general age of the miners has been rising, and will be amenable to proof in future surveys. As primary liver cancer affects men at younger ages than the other sites under consideration (peak age 33·6 years in $t_1$), this too could have contributed towards the decrease in the proportional frequency. The steep and continuous fall in the crude rate (80·5 to 30·8), a 62% decrease over the 16-year period, is one of the most remarkable features of the temporal analysis. The cause of this is not known but it could be due to some basic improvement in general living conditions or to improved food storage resulting in reduction of specific carcinogens or their promoting factors in the diet.

The spatial distribution of primary liver cancer still retains its original distinctive pattern of higher and lower rates in specific areas, both between territories and locally within Mozambique.

Natal remains the only other territory with a crude rate higher than the mean for the whole group.

Little is known of local temporal or spatial fluctuations in incidence of endemic viral hepatitis-B in Mozambique, which may be of importance as a cause, co-factor or result of the induction of primary liver cancer (Kew *et al.*, 1974, 1980; Alexander, 1980).

Although the crude rates for oesophageal cancer are not increasing, there is little doubt that the disease is now occurring more evenly over Transkei itself and also in areas of South Africa where it has not previously been recorded as particularly common. The most noticeable example is to be found in the Orange Free State. The highest rates are now to be found in the Cape, Natal, the Orange Free State and the Transvaal, in that order. Territories outside South Africa have much lower rates.

Oesophageal cancer patients from the Cape accounted for 67% of the total in $t_1$ and dropped to 62·6% in $t_2$, with an overall figure of 65·4% in $t_3$. As indicated, this has happened because the disease is occurring more evenly within Transkei and in areas other than the Cape. It seems that although it was first noticed among the Xhosa of the Cape, it is now occurring in most other black ethnic groups in South Africa.

In $t_1$ the ratio of oesophageal cancer to liver cancer in Cape miners was 1:0·86; by $t_3$ this changed to 1:1·21, reflecting a drop in liver cancer in the Cape, whilst the crude rate for oesophageal cancer remained constant.

Cancer of the respiratory system is on the increase in South Africa, as shown by significantly higher rates in Natal, Transvaal and the Cape, with the Orange Free State having a high, though not significantly high rate. There is also a real increase in these rates since 1964–71 (Harington *et al.*, 1975). Unfortunately, no data on cigarette smoking among the miners are available. The above figures may suggest that the habit may be becoming more extensive, particularly among miners from South Africa itself.

Bladder cancer particularly in Mozambique shows an indication that it may, with primary liver cancer, be showing a real decrease.

The indication of certain sites of cancer occurring with either similar or inverse patterns in the 10 homelands from which the gold miners are recruited is being actively pursued both with other sites of cancer and with data sets from other localities.

We thank Mr C. P. S. Barnard, Chamber of Mines of South Africa, mine managers and senior medical officers of mine hospitals for case records. In particular, Mr A. J. Dekker of the Chamber, and Mr R. T. Faehse of the Employment Bureau of Africa (TEBA), Johannesburg, provided invaluable assistance in making available records of recruits throughout the 16 years of the survey.

### REFERENCES

ALEXANDER, J. J. (1980) Human liver cancer and hepatitis-B virus. *S. Afr. J. Sci.*, **76**, 3.

BRADSHAW, E. HARINGTON, J. S. (1976) Temporal changes in primary liver cancer in black gold miners from Mozambique. *S. Afr. Med. J.*, **50**, 2022.

HARINGTON, J. S., McGLASHAN, N. D., BRADSHAW, E., GEDDES, E. W. & PURVES, L. R. (1975) A spatial and temporal analysis of four cancers in African gold miners from Southern Africa. *Br. J. Cancer*, **31**, 665.

KEW, M. C., GEDDES, E. W., MACNAB, G. M. & BERSOHN, I. (1974) Hepatitis-B antigen and cirrhosis in Bantu patients with primary livei cancer. *Cancer*, **34**, 539.

KEW, M. C., RAY, M. B., DESMET, V. J. & DESMYTER J. (1980) Hepatitis-B surface antigen in tumour tissue and non-tumorous liver in black patients with hepatocellular carcinoma. *Br. J. Cancer*, **41**, 399.